# A longitudinal study reveals persistence of antimicrobial resistance on livestock farms is not due to antimicrobial usage alone

**DOI:** 10.3389/fmicb.2023.1070340

**Published:** 2023-03-14

**Authors:** Richard P. Smith, Hannah E. May, Manal AbuOun, Emma Stubberfield, Daniel Gilson, Kevin K. Chau, Derrick W. Crook, Liam P. Shaw, Daniel S. Read, Nicole Stoesser, Maria Jose Vilar, Muna F. Anjum

**Affiliations:** ^1^Department of Epidemiological Sciences, Animal and Plant Health Agency, Weybridge, United Kingdom; ^2^Department of Bacteriology, Animal and Plant Health Agency, Weybridge, United Kingdom; ^3^Modernising Medical Microbiology Consortium, Nuffield Department of Medicine, John Radcliffe Hospital, University of Oxford, Oxford, United Kingdom; ^4^National Institute for Health Research, Health Protection Research Unit, University of Oxford in Partnership with Public Health England (PHE), Oxford, United Kingdom; ^5^Molecular Ecology Group, UK Centre of Ecology and Hydrology (UKCEH), Wallingford, United Kingdom

**Keywords:** antimicrobial usage, antimicrobial resistance, cattle, sheep, pigs, longitudinal

## Abstract

**Introduction:**

There are concerns that antimicrobial usage (AMU) is driving an increase in multi-drug resistant (MDR) bacteria so treatment of microbial infections is becoming harder in humans and animals. The aim of this study was to evaluate factors, including usage, that affect antimicrobial resistance (AMR) on farm over time.

**Methods:**

A population of 14 cattle, sheep and pig farms within a defined area of England were sampled three times over a year to collect data on AMR in faecal Enterobacterales flora; AMU; and husbandry or management practices. Ten pooled samples were collected at each visit, with each comprising of 10 pinches of fresh faeces. Up to 14 isolates per visit were whole genome sequenced to determine presence of AMR genes.

**Results:**

Sheep farms had very low AMU in comparison to the other species and very few sheep isolates were genotypically resistant at any time point. AMR genes were detected persistently across pig farms at all visits, even on farms with low AMU, whereas AMR bacteria was consistently lower on cattle farms than pigs, even for those with comparably high AMU. MDR bacteria was also more commonly detected on pig farms than any other livestock species.

**Discussion:**

The results may be explained by a complex combination of factors on pig farms including historic AMU; co-selection of AMR bacteria; variation in amounts of antimicrobials used between visits; potential persistence in environmental reservoirs of AMR bacteria; or importation of pigs with AMR microbiota from supplying farms. Pig farms may also be at increased risk of AMR due to the greater use of oral routes of group antimicrobial treatment, which were less targeted than cattle treatments; the latter mostly administered to individual animals. Also, farms which exhibited either increasing or decreasing trends of AMR across the study did not have corresponding trends in their AMU. Therefore, our results suggest that factors other than AMU on individual farms are important for persistence of AMR bacteria on farms, which may be operating at the farm and livestock species level.

## Introduction

The increasing prevalence of antimicrobial resistance (AMR) is currently one of the greatest threats to global public health (World Health Organisation, 2020). AMR increases the costs and decreases the efficiency of healthcare, and with the rise of multi-drug resistant (MDR) bacteria there are concerns that this could lead to greater mortality from common, preventable infections. Although AMR in bacteria occurs naturally in the absence of antimicrobial use, antimicrobial usage (AMU) represents a selection pressure contributing to the proliferation of bacterial AMR through mutation and transfer of mobile genetic elements (e.g., plasmids) between bacteria (AbuOun et al., 2020). AMU in livestock in Europe was previously more extensive, and antimicrobials were administered for non-therapeutic, growth-promoting purposes as well as clinical reasons. However, since 2006, non-therapeutic use has been banned within the European Union (Cogliani et al., 2011) and organic farming, which prohibits non-therapeutic antimicrobial use, has become more popular (European Parliament, 2015).

Studies have found significant associations between AMU and AMR in livestock and human populations at the individual person or animal, farm or hospital level, and in larger population-scale studies (Tan et al., 1992; Bronzwaer et al., 2002). In a previous cross-sectional study in the UK in slaughtered pigs, increased AMR was significantly associated with medium or high categories of AMU on the source farms (AbuOun et al., 2020). Studies have also shown that reducing AMU can lead to relatively quick reductions in AMR; in a study of a farm experiencing post-weaning diarrhea from which colistin-resistant bacteria were detected, the cessation of colistin use was followed by a reduction of resistant bacteria in ~3.5 months (Randall et al., 2018). However, low usage of antimicrobials on farms can still promote presence of a low-level persistent bacterial population harbouring AMR (Storey et al., 2022). Studies of Finnish urinary tract infection patients and *Campylobacter* in Canadian sheep farms found that most AMU-AMR associations were not statistically significant (Bergman et al., 2009; Scott et al., 2012). In other studies, co-selection has been detected, where AMU of one antimicrobial class has been associated with AMR to another class (Akwar et al., 2008). Therefore, the associations between AMU and AMR have not been demonstrated consistently (European Centre for Disease Prevention and Control (ECDC), European Food Safety Authority (EFSA) and European Medicines Agency (EMA), 2021).

AMR has also been shown to be associated with other factors, some of which may be proxies for AMU. In a US study of cattle farms, MDR in *Escherichia coli* was associated with geographic region, animal age, farm type (Berge et al., 2010). Two separate studies of European pig farms indicated that MDR was positively associated with farms which did not apply complete all-in-all-out systems, the presence of lameness, ill-thrift or shoulder scratches, and sampling in the summer months (June to August), whereas samples from farms that never cleaned and disinfected finisher pens, and those that fed pigs from the floor and/or fed coarsely ground pig feed were significantly less likely to have MDR (Schuppers et al., 2005; AbuOun et al., 2020). In a review of 28 studies of *Campylobacter*, *E. coli*, and *Salmonella* in beef cattle, broilers, and pigs, AMU was a significant association for AMR presence in nearly half of studies, whereas associations with farm management type (e.g., organic, conventional) and feed practices were less commonly detected (Murphy et al., 2018).

Although the relationship between AMU and AMR can be complex, there is concern that AMU in livestock will lead to a reservoir of AMR bacteria that could reduce the effectiveness to treat animal illness, and also could transfer this risk to the public health field *via* the food chain and/or environment. Due to this, UK initiatives have been enacted to reduce this threat. AMU in animals is risk stratified (Responsible Use of Medicines in Agriculture, 2020) to limit the use of treatments that are critically important to human health (Highest Priority Critically Important Antibiotics (HP-CIAs)). There are also schemes in place to collect and monitor annual AMU and AMR, to assist farmers and veterinary practitioners in reducing AMU on farm (Veterinary Medicines Directorate, 2020).

We previously undertook a study evaluating AMU and AMR on 14 livestock farms at a single time point, which highlighted that differences in AMR gene and AMR plasmid carriage varied with the host animal and there were statistically significant associations between AMU and AMR in *E. coli* (AbuOun et al., 2021). Here, we extended this work by longitudinally evaluating changes in AMU and other farm factors in the same 14 farms, alongside AMR gene presence in multiple bacterial species derived through whole genome sequencing (WGS). The focus of this study was to concentrate on the epidemiological aspects of AMR using WGS and other data.

## Materials and methods

### Farm recruitment and data collection

We aimed to recruit five farms for each of three livestock species [pig, sheep and cattle (dairy and beef)] within a defined geographical area (south central England). All farms within the area were identified, and those with the largest herd sizes invited to join the study, to ensure commercial-sized farms were recruited. Five cattle and sheep farms provided consent to join the study (see supplementary material for example of consent form) although only four pig farms could be recruited due to the lack of available pig farms willing to participate, leaving a population of 14 farms (Table 1). Full details of the farms and their recruitment are provided in the preliminary cross-sectional study (AbuOun et al., 2021).

**Table 1:** Summary of genotypic antimicrobial resistant classes (AMR) and multi-drug resistant (MDR) isolates detected from isolates of fecal Enterobacterales collected from 14 livestock farms from three sampling points within a year.

Animal species	Farm type	Total no. of isolates	Mean no. AMR classes per isolate (range)	Most common AMR profile (no. isolates)	No. MDR isolates	Most common genotypic MDR profile (no. isolates)^*^
Cattle	4 dairy, 1 dairy & beef finisher	174	0.24 (0–8)	tetra (7)	5	Amino, betaL, trime, sulph, tetra (2)
Pigs	2 farrow-to-finish, 1 finisher, 1 grow-to-finish	140	2.70 (0–8)	tetra (19)	65	Amino, betaL, phen, trime, sulph, tetra (14)
Sheep	4 lowland, 1 hill	170	0.22 (0–5)	tetra (9)	6	Amino, betaL, trime, sulph, tetra (5)
Total	-	484	0.95 (0–8)	tetra (35)	76	Amino, betaL, trime, sulph, tetra (15)

The summary compares the average and range of resistances detected to antimicrobial classes and the most common AMR and MDR profiles detected between the three animal species sampled.

*Amino, aminoglycoside; betaL, beta-lactam; phen, amphenicol; sulph, sulphonamide; tetra, tetracycline; trime, trimethoprim.

Each farm was visited on three seasonal occasions in 2017. Winter visits occurred between January and April, summer visits between June and July and autumn visits October to November. Individual visits to each farm were planned to allow at least 3 months between them.

Each farm was split into up to five epidemiological groups, representing animals managed in the same way (e.g., animals of the same age, housed in the same building). At each visit, 10 pooled fecal samples were collected, with each pooled sample made up of 10 small pinches of fresh feces collected from the floor using a gloved hand and combined to form a golf ball-sized sample. The 10 pooled samples were then pooled again to create a single composite sample per farm. The sampling locations were chosen to be representative of the farm, with roughly two samples per epidemiological group, although this was adjusted where less than five groups were present or a disproportionate number of animals occurred in each group. When collecting samples from each group the samples were taken from different locations to be representative, e.g., in a building of 20 pens, every second pen had a pinch taken from it, or fields were split into sectors and pinches taken from each sector.

At each visit a group-level record was completed, characterizing weather conditions at the time of sampling, the number and type of animals present in each group and details of their housing types or pasture locations. Additionally, a farm-level questionnaire was completed by farmers directly before the first sampling visit to capture farm structure and management information, such as hygiene, disinfection, and biosecurity practices. Any relevant changes to the questionnaire topics were noted by sampling staff at subsequent farm visits. AMU in the preceding 3 months was also documented for all farms, with details collected on products used and type and number of animals treated. Additionally, two pig farms gave consent for access to their electronic Medicine Book (eMB) (Agriculture and Horticulture Development Board, 2020), recording all veterinary medicinal product use (gross quantities/quarter). Three cattle farms also facilitated access to herd management software where all medicinal use was recorded at the individual animal level.

### Sample testing and whole genome sequence analysis

Faecal samples were diluted in buffered peptone water, plated on to CHROMagar™ ECC (CHROMagar Microbiology, Paris, France) and 14 colonies were selected. MALDI-TOF (Bruker, Coventry, UK) or 16S rRNA sequencing (Edwards et al., 2012) was used to determine the bacterial species present. Up to 14 isolates per visit were sent for WGS, targeting *Escherichia* spp., *Citrobacter* spp. and *Klebsiella* spp.

For these isolates, genomic DNA was extracted, Illumina short read sequencing and Oxford Nanopore Technology MinION or PacBio long read sequencing was performed (between 9 and 14 per farm per time point), as well as sequence data assembled for further analysis as described in AbuOun et al., 2021 and Shaw et al., 2021. Sequencing had already been completed on isolates from time point 1 (as detailed in AbuOun et al., 2021), but new sequencing was performed on isolates from time points 2 and 3. Kraken was used to confirm bacterial species (Wood and Salzberg, 2014), using short read sequencing data. The AMR gene content of isolates was determined from short read sequencing data using the APHA SeqFinder pipeline^1^ and corroborated with the hybrid assembles, using Abricate^2^. A binary resistance classification for each antimicrobial class inferred on the basis of genotype (Duggett et al., 2017; Stubberfield et al., 2019). AMR genes known to be intrinsic to *Citrobacter* spp. and *Klebsiella* spp. were excluded (Appendix Table A).

### Data analysis

Questionnaire data were cleaned prior to analysis, with missing values replaced by values of “not known” or “not applicable.” The total weight of each active antimicrobial ingredient (mg) was calculated, to allow the determination of milligrams per kilograms (mg/kg) of animals treated. European standardized animal weights were used to determine the estimated average kg weight of each animal type named as being treated by each product at time of treatment (European Medicines Agency, 2011). These calculations required assumptions to be made in order to correct areas where data was missing or was unable to be distinguished, as explained previously (AbuOun et al., 2021). Each active ingredient was also grouped at the antimicrobial class-level for ease of comparison to the AMR profiles detected (Magiorakos et al., 2012).

The AMU data and the genotype results for each antimicrobial class were summarized per farm and per visit. Isolates were classified as MDR, if they had genotypic resistance to more than two antimicrobial classes (Magiorakos et al., 2012). The AMR results (% isolates resistant) were summarized at the farm-level and at the bacterial species level to allow classical multi-dimensional scaling (MDS) to summarize the similarity of the results through two dimensions and provide an easy to interpret comparison, which were plotted using Minitab 16 (Minitab inc.).

The farm and group level data collected at each visit were assessed to detect any changes in management and these ‘change’ variables were combined with the AMU data and AMR results and imported into STATA 16 (StataCorp, 2020. Stata Statistical Software: Release 12. College Station, TX: Stata-Corp LP) for analysis. A mixed-effects logistic model was used to identify significant risk factors (*p* < 0.05) associated with an isolate being MDR. The unique farm identifiers (recorded as RH01-RH15) were included in the model as a random effect to account for farm-level clustering. An initial univariable screening stage was used to assess all independent variables, with those with a *p*-value>0.25 omitted from further analysis. A backwards stepwise method was then used to select variables to be retained in the model. At each step variables with the greatest p-value were removed from the model and a Likelihood Ratio test was used to determine whether the removal significantly improved the fit of the model. The final multivariable model was fitted from variables retained in the model that had a Likelihood Ratio *p*-value<0.05. Where variables were collinear (e.g., month of visit and season of visit), after univariable assessment, only the best fitting variable was progressed to the multivariable step.

## Results

### Description of isolates by bacterial species

Table 1 provides details of the 14 recruited farms. Further details on these farms can be found in AbuOun et al. (2021). A total of 484 isolates were sequenced over the three visits to each farm, with a mean of 11.5 (range 9–14) isolates per farm per visit. Most isolates were *E. coli* (389, 80.4%) or *E. fergusonii* (51, 10.5%) but other *Escherichia* spp. (2, 0.4%) were identified as well as *Citrobacter* spp. (31, 6.4%) and *Klebsiella* spp. (11, 2.3%) (Appendix Table B). AMR genes were detected by the APHA SeqFinder. No AMR genes were detected in 355 (73.3%) isolates, but in the remaining isolates genotypic resistance was detected for up to eight different AMR classes. Forty-one isolates had resistance to one antimicrobial class detected (8.5%), 12 had two (2.5%), 9 had three (1.9%), 10 had 4 (2.1%), 25 had 5 (5.2%), 25 had 6 (5.2%), 5 had 7 (1.0%) and 2 had 8 (0.4%). In total, 76 isolates were identified as MDR (15.8%).

Multi-dimensional scaling summarising the similarity of AMR genotypes amongst bacterial species suggested that most *Escherichia, Citrobacter, and Klebsiella* isolates had relatively similar AMR genotypes, whereas the single isolates of *Escherichia marmotae* and *Klebsiella oxytoca* were dissimilar (Appendix Figure A).

### Antimicrobial resistance by animal species

AMR genes were detected by the APHA SeqFinder and the genotypic results have been summarised by their AMR class (Appendix Table C). The mean number of AMR classes, in which genes conferring resistance to a particular AMR class were detected, differed between animal species (Table 1). More were detected on average in pigs, than cattle or sheep, as seen previously in our study evaluating the first time point (AbuOun et al., 2021). The farms with the greatest average number of isolates with any AMR genes were two pig farms: RH01 and RH02 (3.9 and 5.1 classes respectively). More AMR isolates were detected from the pig farms per visit than from the other species (Table 1), at all three timepoints. Tetracycline monoresistance was the most common AMR class profile observed across all three animal species at all visits.

The most common MDR profile combined aminoglycoside, beta-lactam, trimethoprim, sulphonamide and tetracycline resistance genes. This was also the most common AMR profile in isolates from cattle and sheep farms, whereas the most common MDR isolates in pigs also included additional amphenicol resistance genes (Table 1). MDR was detected in 54 (13.9%) *E. coli*, 12 (23.5%) *E. fergusonii*, 7 (22.6%) *Citrobacter* spp., and 3 (27.3%) *Klebsiella* spp. We observed a wide diversity of AMR profiles at the class level in the 129 genotypically resistant isolates, with 8, 26 and 3 different profiles in isolates from cattle, pigs, and sheep, respectively.

**Figure 1:**
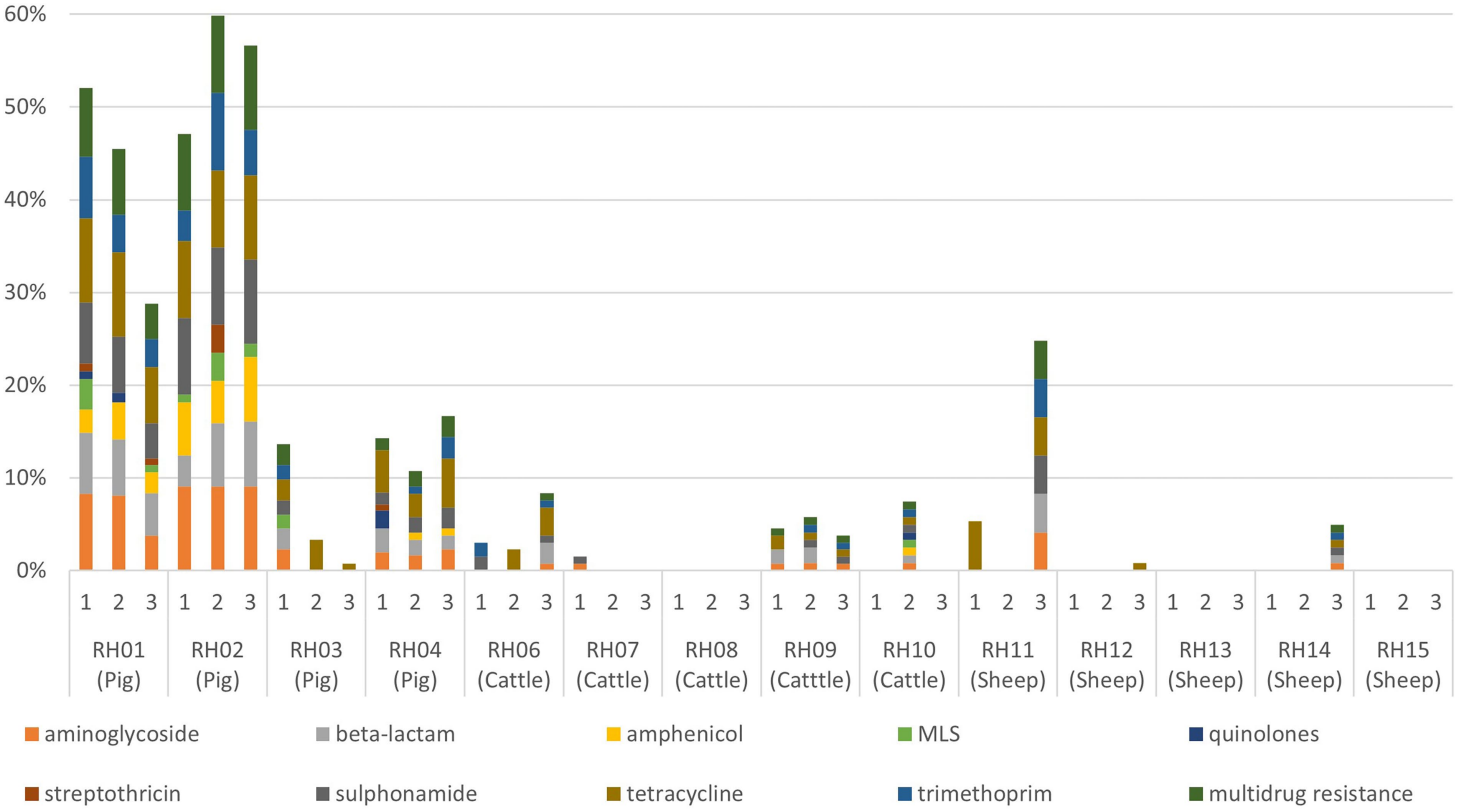
Proportion of faecal Enterobacterales isolates resistant to each antimicrobial resistance class (and multidrug resistance) for each visit to 14 livestock farms (RH01–RH15). Pig farms were farms RH01-RH04, cattle farms were RH06-RH10, and sheep farms were RH11–RH15. MLS, macrolides, lincosamides, and streptogramins.

AMR genes were infrequently detected in isolates from visits to the sheep farms (Table 2; Figure 1). For cattle, there were slightly fewer visits that had any AMR genes for any antimicrobial detected in isolates than was identified from pig farm visits, and the proportion of AMR positive isolates detected in total was consistently lower than from pigs. The patterns of resistance detected at each visit for a farm was relatively consistent, with some interesting exceptions such as farms RH03 and RH11. These farms had visits in which resistance to five or more classes detected at a single visit, whereas other visits produced either fully susceptible or only tetracycline-resistant isolates (Figure 1).

**Table 2:** Mean proportion of faecal Enterobacterales isolates, from three sampling visits to three different livestock species (14 farms in total), that harboured resistance to each antimicrobial class, with standard deviation (St.Dev.) provided.

Animal species	Results	Antimicrobial resistance class									
		amino	betaL^a^	phen	MLS	quino	strep	sulph	tetra	trime	MDR
Pigs	Mean % isolates	50.9%	38.8%	25.4%	9.9%	3.5%	4.8%	44.8%	62.9%	31.9%	47.2%
	St.Dev.	41.4%	28.7%	27.3%	13.2%	6.9%	9.7%	37.5%	33.5%	29.8%	38.4%
	Visits with AMR isolates detected										
	No.	10/12	10/12	8/12	6/12	3/12	4/12	10/12	12/12	9/12	10/12
	%	83.3%	83.3%	66.7%	50.0%	25.0%	33.3%	83.3%	100.0%	75.0%	83.3%
Cattle	Mean % isolates	3.4%	4.6%	0.6%	0.6%	0.6%	0.0%	4.0%	6.8%	3.4%	2.9%
	St.Dev.	4.4%	8.4%	2.3%	2.3%	2.3%	0.0%	5.4%	10.6%	5.4%	4.2%
	Visits with AMR isolates detected										
	No.	6/15	4/15	1/15	1/15	1/15	0/15	6/15	6/15	5/15	5/15
	%	40.0%	26.7%	6.7%	6.7%	6.7%	0.0%	40.0%	40.0%	33.3%	33.3%
Sheep	Mean % isolates	3.6%	3.6%	0.0%	0.0%	0.0%	0.0%	3.6%	8.1%	3.6%	3.6%
	St.Dev.	11.8%	11.8%	0.0%	0.0%	0.0%	0.0%	11.8%	18.2%	11.8%	11.8%
	Visits with AMR isolates detected										
	No.	2/15	2/15	0/15	0/15	0/15	0/15	2/15	4/15	2/15	2/15
	%	13.3%	13.3%	0.0%	0.0%	0.0%	0.0%	13.3%	26.7%	13.3%	13.3%
All	Mean % isolates	17.1%	14.0%	7.5%	3.1%	1.2%	1.4%	15.5%	23.3%	11.6%	15.8%
	St.Dev.	31.3%	23.3%	18.3%	8.3%	4.1%	5.5%	28.0%	33.1%	21.5%	29.2%
	Visits with AMR isolates detected										
	No.	18/42	16/42	9/42	7/42	4/42	4/42	18.42	22/42	16/42	17/42
	%	42.9%	38.1%	21.4%	16.7%	9.5%	9.5%	42.9%	52.4%	38.1%	40.5%

The numbers of visits to a farm have also been combined for each animal species to determine percentage of visits where AMR genes from a particular class were detected in isolates.

amino, aminoglycoside; betaL, beta-lactam; phen, amphenicol; MLS, macrolides; lincosamides, and streptogramins; quino, quinolones; strep, streptothricin; sulph, sulphonamide; tetra, tetracycline; trime, trimethoprim; MDR, multidrug resistance.

a All non-ESC beta-lactam apart from 3 ESC beta-lactam resistant isolates (from 2 cattle farms and 1 pig farm).

The standard deviations of the proportion of isolates with AMR were consistently greater than the mean for cattle isolates for all antimicrobial classes (apart from streptothricin resistance, which was all negative for cattle), indicating larger variation in the proportion of resistant isolates per farm visit (Table 2). The summary results from pig farms indicated more stability in the presence of AMR-harboring isolates between farms and visits compared to other livestock, with generally a lower standard deviation proportional to the mean. The unsummarized results from each farm visit are presented in Appendix Table D.

Multi-dimensional scaling summarizing the isolate AMR genotype profiles by farm showed a tight cluster of farms (cattle farms RH07, RH08 and RH10, sheep farms RH12-15) with few AMR isolates (range 0–3) and only 0–1 MDR isolate per visit (Appendix Figure B). Pig farm RH03 and cattle farm RH06 were also determined to be similar and both of their AMR results indicated a small (<4) number of isolates with MDR present at a single visit and small number (1–4) of isolates with one or two resistances at other visits. Notably pig farms RH01 and RH02 were dissimilar to the other farms, reflecting their high proportion of MDR isolates (66% of isolates from RH01, 94% of isolates from RH02). MDR Enterobacterales isolated from these farms harbored one or two plasmid-mediated quinolone resistance (PMQR) genes encoding resistance to quinolones or macrolide resistance gene (*mphAB*/ *mefB*) (17% from RH01 and 58% from RH02). Quinolones are listed as a HP-CIA, and macrolides are an WHO HP-CIA, although not classed as such by the European Medicines Agency. These genes were harbored in addition to commonly detected AMR genes, such as those conferring sulphonamide and tetracycline resistance. Also, a significantly higher proportion of *E. coli* from RH02 (11/19, 58%) harbored PMQR plasmids or MLS (macrolides, lincosamides, and streptogramins) resistance compared to *E. coli* from RH01 (5/24, 21%; Fisher’s Exact Test *p* < 0.001).

### Antimicrobial usage

**Figure 2:**
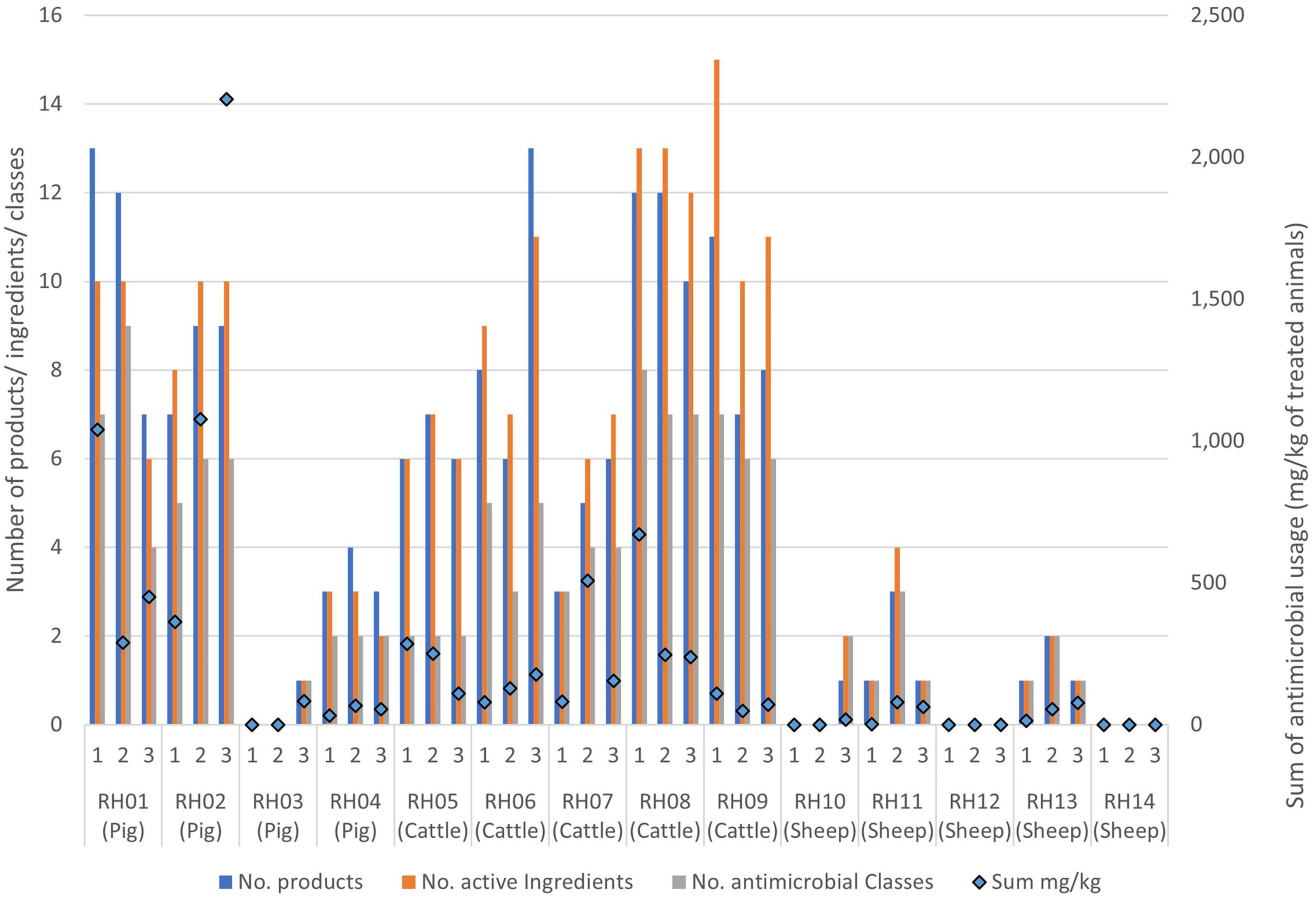
Antimicrobial usage on the 14 livestock farms collected from three separate visits to each farm. Usage is presented as data bars displaying the number of unique antimicrobial products, unique active ingredients, and antimicrobial classes used per visit. Points represent the sum of mg of active ingredients per kg of treated animal weight used prior to each visit.

The most commonly used antimicrobial classes were the non-extended spectrum cephalosporins (non-ESC), with usage reported in the 3 months prior to 26/42 (62%) farm visits, and aminoglycosides, with use reported prior to 22/42 (52%) visits. Total AMU over three 3-month periods preceding all three visits indicated that pigs and cattle on average used the most amount (in weight) of different antibiotic products, active ingredients, and antimicrobial classes in total per farm, when compared to sheep (Table 3; Figure 2). The total AMU on pig farms RH01 and RH02 was substantially larger than the other farms, but when converted to mg/kg of treated animals, the values were similar to the highest-usage cattle farm RH09 (Table 3). There was greater diversity of antibiotic products used amongst pig farms (ranging from 1 to 24 different products per farm over the study period) when compared to cattle, which were more consistent in the products used between farms and visits (8–19 products per farm). The pig farms also used more group treatments (in-feed or in-water) than individual treatments, which equating to 87% of AMU (in mg/kg of treated animals) on pig farms, whereas group treatments were not used on sheep farms and only an average of 7.5% of AMU was intramammary treatment on the cattle farms.

**Table 3:** Summary of antimicrobial usage on 14 livestock farms reported as used in the 3 months before three farm visits over a period of 12 months (i.e., covering 9  months).

Farm ID	Species	No. products	No. active Ingredients	No. antimicrobial Classes	Total quantity (mg)	Sum mg/kg of treated animals
RH01	Pig	24	16	9	203,592,400	1,779
RH02	Pig	11	11	7	138,402,625	3,643
RH03	Pig	1	1	1	5,400	83
RH04	Pig	5	3	2	252,405	152
RH06	Cattle	8	7	2	2,642,582	644
RH07	Cattle	17	14	6	665,960	384
RH08	Cattle	10	12	6	306,430	743
RH09	Cattle	19	16	9	2,419,246	1,155
RH10	Cattle	13	15	7	93,990	228
RH11	Sheep	1	2	2	1,350	18
RH12	Sheep	4	4	3	53,500	144
RH13	Sheep	0	0	0	0	0
RH14	Sheep	3	2	2	127,400	145
RH15	Sheep	0	0	0	0	0

The summary details the number of unique antimicrobial products [i.e., those listed in the NOAH compendium (www.noahcompendium.co.uk)], active antimicrobial ingredients and antimicrobial classes used over the period on each farm, as well as the total quantity (in mg) of active ingredients used in that period and modified by the estimated weight of the treated group of animals for each treatment.

Generally, the number of antimicrobial classes used was relatively consistent across the three visits for each farm, varying by only one added or removed class across visits for four of the cattle farms (RH06 and RH08-RH10), three sheep farms (RH13-RH15) and three pig farms (RH02-RH04) (Figure 2; Appendix Table E). A further two sheep farms (RH11 and RH12) and one cattle farm (RH07) deviated by two products during the study. Finally, the remaining pig farm (RH01) deviated by five products (4–9 antimicrobial classes per visit). However, the quantity of the products differed substantially between visits, with two pig farms (farms RH01 and RH02) using 79 and 64% of their total recorded AMU in a single visit (at visits 1 and 3 respectively). Additionally, one sheep and one pig farm only used antimicrobials at a single visit. In contrast, five farms (one pig and four cattle farms) had less than 50% usage before each single visit, indicating a more even spread of AMU across the visits, even if the specific classes used differed over time.

**Figure 3:**
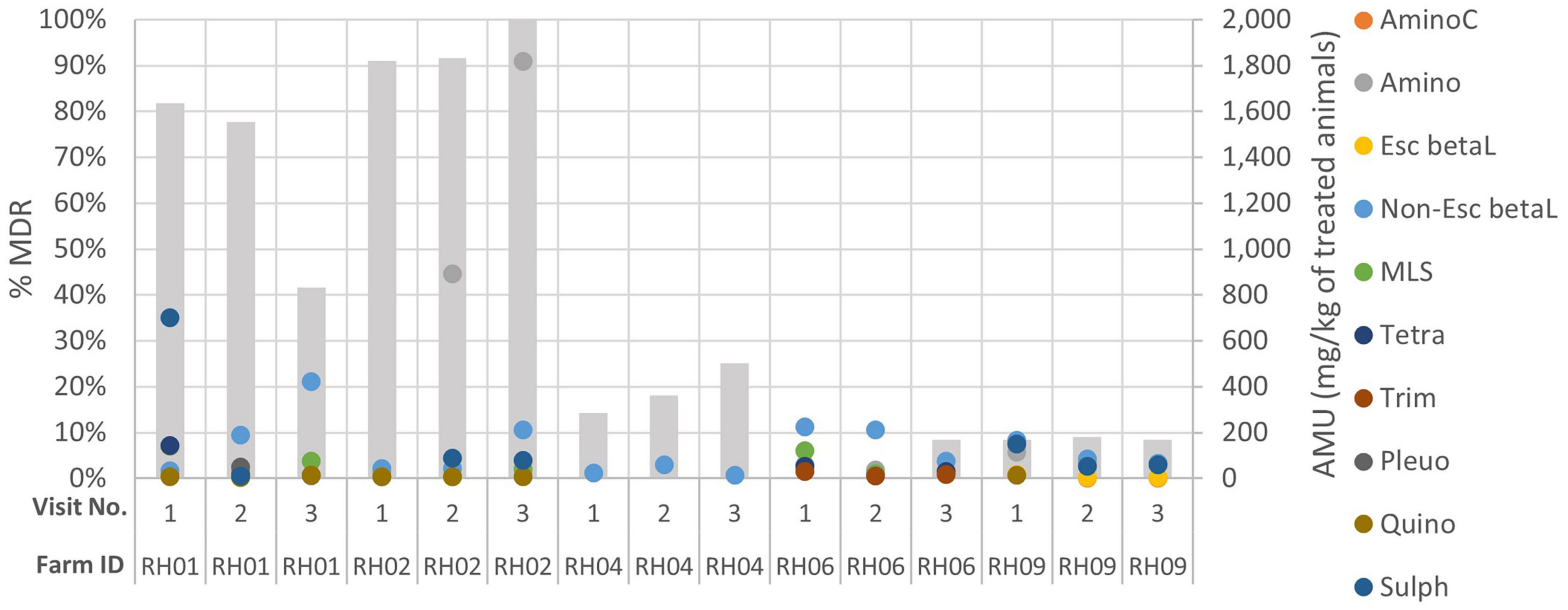
Comparison of genotypic multi-drug resistance (MDR; represented by the grey columns) detected in isolates and antimicrobial usage (AMU) by class (in mg of active ingredient/kg of estimated weight of the treated group of animals for each treatment) on five livestock farms (RH01, RH02, RH04, RH06 and RH09) which had demonstrated increasing or decreasing AMR trends across their three sampling visits (labelled 1–3). AminoC, aminocoumarin; Amino, aminoglycoside; Esc betaL, ESC beta-lactam; non-Esc beta, non-ESC beta-lactam; MLS, macrolides, lincosamides, and streptogramins; tetra, tetracycline; trime, trimethoprim; Pleuro, pleuromutilin; Quino, quinolone; Sulph, sulphonamide.

When the AMU was compared to AMR for each farm over the three visits, decreasing or increasing resistance trends to at least one antimicrobial class were visualized for five farms: three pig and two cattle farms. These farms were assessed to explore the patterns of AMU and AMR for specific antimicrobial classes (Figure 3; Appendix Figures C–G); however, no consistently clear visual association between AMU and AMR was seen for any farm over the visits.

Interestingly, three farms had AMR isolates detected at only a single visit but had reported AMU throughout the study (Appendix Figures H–J). Cattle farm RH07 had a single isolate detected which harbored aminoglycoside and sulphonamide resistance at visit 1, followed by a rise in quantity of AMU (including aminoglycoside use) at visits 2 and 3 - but no resistant isolates were detected at visit 2 and 3. Cattle farm RH10 only had resistance detected at visit 2, with one MDR isolate resistant to seven AMR classes (including ESC beta-lactam). This was despite total AMU on this farm being reduced from 109 mg/kg to 47 mg/kg of treated animals at visit 2. Sheep farm RH12 only showed resistance to a single antimicrobial class (tetracycline) at visit 3, which coincided with a steady increase in tetracycline use at visits 2 and 3.

### Statistical analysis of associations

The risk factor model for MDR outcomes assessed the presence of significant association of MDR bacteria with 12 husbandry/ management or seasonal conditions variables and 12 AMU variables (including sum of all AMU in mg/kg of treated animals and number of antibiotic classes used). Although 14 variables progressed from the univariable screening stage (*p* < 0.25), and 6 were significant (*p* < 0.05) at this stage, only a single variable was retained in the final multivariable risk factor model, which was the number of unique antibiotic classes used in the 3 months before a visit (Table 4). The amount of variation explained by this model was estimated to be 15% (pseudo-*R*^2^). For each additional antibiotic class used, the risk of an isolate being MDR increased by 1.6 (*p* = 0.002).

**Table 4:** Univariable mixed-effects models detailing individual associations between genotypic multi-drug resistance (MDR), detected in faecal Enterobacterales isolates (*n* = 484) and farm management factors.

Variable	Category	mean (SD) variable response for positive isolates^†^	mean (SD) variable response for negative isolates^†^	No. isolates MDR Positive^a^	No. isolates MDR Negative^a^	% MDR Pos.	Odds Ratio	*p*-value	95% confidence interval	
Month of sampling	Jan			19	15	55.90%	1.649	0.582	0.277	9.806
	Feb			0	58	0.00%	Predicts failure perfectly			
	Mar			4	53	7.00%	5.037	0.126	0.635	39.95
	Apr			2	12	14.30%	0.752	0.836	0.051	11.129
	Jun			12	78	13.30%	Baseline			
	Jul			10	56	15.20%	1.6	0.662	0.195	13.152
	Oct			20	75	21.10%	5.854	**0.032**	1.168	29.335
	Nov			9	61	12.90%	0.844	0.874	0.103	6.892
Season of sampling	Winter			28	134	17.30%	Baseline			
	Spring			2	12	14.30%	0.732	0.747	0.11	4.88
	Summer			22	134	14.10%	1.326	0.586	0.48	3.658
	Autumn			24	128	15.80%	6.365	**0.007**	1.657	24.446
Weather at visit	Dry			66	267	19.80%	Baseline			
	Wet			10	141	6.60%	0.388	**0.045**	0.154	0.979
Temperature at visit (°C)	Continuous	20.80 (4.81)	14.12 (6.03)				1.093	0.054	0.998	1.197
Any sampled animals outdoors	No			50	68	42.40%	Baseline			
	Yes			26	340	7.10%	0.286	0.087	0.068	1.201
Any access of animals to farm waste	No			76	373	16.90%	Baseline			
	Yes			0	35	0.00%	Predicts failure perfectly			
Change in public access to farm	No			71	322	18.10%	Baseline			
	Yes			5	86	5.50%	11.796	**0.03**	1.275	109.164
Change in the spreading of farm waste	No			65	365	15.10%	Baseline			
	Yes			11	43	20.40%	1.596	0.373	0.57	4.467
Change in the use of waste brought onto site	No			76	385	16.50%	Baseline			
	Yes			0	23	0.00%	Predicts failure perfectly			
Min. cleanliness score of animals	Continuous	3.24 (1.15)	3.33 (0.95)				1.711	0.126	0.86	3.404
Max. cleanliness score of animals	Continuous	4.41 (0.49)	4.27 (0.60)				2.384	0.052	0.991	5.734
Mean cleanliness score of animals	Continuous	3.79 (0.86)	3.83 (0.64)				2.778	**0.027**	1.121	6.885
Sum of all AMU	Continuous	784.11 (752.12)	118.65 (169.67)				1.002	0.058	1	1.003
Aminocoumarin use	Continuous	0.02 (0.14)	0.13 (0.39)				0.568	0.615	0.063	5.134
Aminoglycoside use	Continuous	452.43 (695.07)	27.87 (65.83)				1.002	0.184	0.999	1.004
ESC beta-lactam use	Continuous	0.89 (2.68)	2.68 (4.62)				1.002	0.975	0.875	1.147
MLS use	Continuous	50.02 (49.29)	13.54 (27.42)				1.004	0.474	0.993	1.015
non-ESC beta-lactam use	Continuous	105.88 (113.43)	47.28 (81.62)				0.997	0.114	0.992	1.001
Pleuromutilin use	Continuous	4.47 (14.12)	0.24 (3.39)				1.019	0.315	0.982	1.058
Quinolone use	Continuous	5.88 (3.95)	1.57 (4.73)				0.936	0.376	0.807	1.084
Sulphonamide use	Continuous	113.59 (219.8)	10.68 (55.60)				1.002	0.115	0.999	1.005
Tetracycline use	Continuous	28.17 (48.55)	12.40 (23.65)				1.007	0.434	0.99	1.024
Trimethoprim use	Continuous	22.77 (43.95)	2.25 (11.24)				1.01	0.115	0.997	1.024
No. AM classes used	Continuous	5.2 (2.31)	2.56 (2.45)				1.635	**0.002**	1.203	2.223

The isolates were collected from 14 cattle, sheep, and pig farms, visited three times over a 12 month period. All antimicrobial usage (AMU) is recorded as mg/kg of treated animals. Farm ID was added as a random effect to account for clustering related to multiple sample visits to the same farms. Bold *p*-values indicate those that are significant (*p* < 0.05).

a Continuous variables are presented as mean (standard deviation (SD)) for positive and negative isolates. MLS = macrolides, lincosamides, and streptogramins.

## Discussion

This study has provided important evidence on the associations between AMR and AMU and other factors that may lead to presence and persistence of AMR bacteria on livestock farms. The results support findings from the previous, linked study which assessed mainly at *E. coli* isolates collected at the first time point (AbuOun et al., 2020). Assessing data at a single time point, did not allow for the analysis of changes in AMU over time, seasonality or other such factors. This study, where on-farm data from three time points and a much larger dataset of Enterobacterales isolates were included, showed conclusively that AMR was detected more frequently on pig farms than other livestock farms, despite two of the pig farms using a relatively low mg/kg of antimicrobials throughout the study. The longitudinal nature of the study allowed for the assessment of the consistency of AMU and detection of AMR between visits to the same farm, and the impact of increasing or decreasing trends of AMU on AMR.

Significant linear associations were not detected between AMU and on-farm AMR by antimicrobial class, at the isolate level. This may relate to differences in the mechanisms of resistance for specific antimicrobials within each class, e.g., apramycin use may not have correlated with aminoglycoside resistance. The investigations of those farms which exhibited either increasing or decreasing trends of AMR across their visits did not have corresponding trends in their AMU. Additionally, a cattle farm (RH09) which presented relatively high AMU (3^rd^ highest overall of mg/kg of treated animals) did not have the same degree of AMR as the two high usage pig farms, whereas the pig farms with low AMU had moderate proportions of AMR detected. These results therefore suggest that factors other than AMU alone are influencing AMR on farms, and that these may be operating at the farm and livestock species level.

AMR isolates were detected consistently across pig farms at all visits. The study results may be explained by a complex combination of factors including: historical AMU within pig populations; co-selection of AMR bacteria, e.g., through the presence of MDR plasmids, particularly on the sampled pig farms (AbuOun et al., 2021); persistent environmental reservoirs of AMR bacteria; or importation of livestock and thus microbiota, from farms supplying pigs to these units. The comparatively short lifespan and high turnover of animals on pig farms compared to cattle farms may increase the risk of introducing diseases which could initiate outbreaks and require widespread treatment and may also impact upon the herd-immunity to resist diseases.

Pig farms may also be at increased risk of AMR due to the greater use of oral routes of medication and group treatment. Medication in pigs, which is frequently given at the group level, often contrasts with treatment of cattle which generally included injectable and intra-mammary products targeted to the individual diseased animal(s). The difference in AMU by livestock species is supported by AMU monitoring in the year of this study (2017) across the UK, with only 3% of active ingredients from pig farms being injectables compared to 69% on cattle farms (Veterinary Medicines Directorate, 2018). Additionally, the AMU of pig farms RH01 and RH02 demonstrated very high peaks of usage recorded within the period before a single visit, which suggested they may have had outbreaks of animal illness.

Although the risk factor model identified a number of variables significantly associated with MDR presence, only one was retained in the multivariable model. The number of different antibiotic classes used by a farm in the 3 months before a visit was a risk factor for the presence of MDR. The fact that this was the key explanatory variable, precluding the addition of other variables into the model, was consistent with the hypothesis that using multiple antimicrobial classes at a location increases the presence of MDR in bacteria at that location. However, it should be noted that the sampled animals were not always the treated animals and this may have affected some ability to detect associations.

Although the study was designed to aid comparison between the results from different farms over the period of 1 year, it should be noted there was some difference in the diversity of selected bacterial species detected from the different farms and visits. However, the majority of isolates from each farm were *E. coli*. The MDS plot suggested that there were reasonable similarities between AMR results from the different bacterial species. *Escherichia marmotae* and *Klebsiella oxytoca* showed some dissimilarity but the results came from few isolates. These findings relate to our previous study, which included some of the isolates reported here and demonstrated that the bacterial chromosomes clustered by genus, but the plasmid pangenomes were more diverse and overlapping between genus (Shaw et al., 2021). The isolates sequenced for this study were also not selected at random, but chosen to ensure a representation of diverse Enterobacterales that were present. This selection may not have been representative of the composition of the bacterial population present in each sample. Future studies using metagenomics will help determine whether the effects of AMU and other factors on AMR-harboring Enterobacterales can be extrapolated to the resistome. We acknowledge that the study population was relatively small, with only few farms for each livestock species and farm management type. Farms were also only selected from a single geographical region, so while the results make sense in the context of what is known about AMR, they may not generalize to other settings, particularly in regions with different climates or animal husbandry practices.

The calculation of mg/kg of treated animals for AMU was necessarily based on standardized weight classifications and other such assumptions, such as those used in the event of missing information. Therefore, this measure may not accurately represent the AMU per kg of animal treated. However, it provided a useful, standardized value to compare AMU between the species. Antimicrobial use that occurred before the period that AMU data was recorded for this study may also have impacted on the presence of AMR genes detected during analysis. Longer timescales may be needed to study the relationship between AMU and AMR. It should also be noted that the recorded AMU was not specific to the sampled groups, but applied across farm, and so may have lowered the ability to detect true associations. The study identified AMR through genotypic methods which might not fully reflect phenotypic resistances expressed, and the authors accept that new resistance genotypes would not have been identified. However, there is now a large body of work which demonstrates that there is very high correlation between geno- and pheno-types and that AMR can be inferred from presence of the corresponding genetic determinant (e.g., Stubberfield et al., 2019; Bortolaia et al., 2020; Davies et al., 2020; Storey et al., 2022).

In conclusion, this study has provided an assessment of the multiple factors that affect the presence of AMR in bacteria on sheep, cattle, and pig farms over time. The results support our previous analysis taken from a single time point: on-farm AMU is associated with on-farm AMR but cannot completely explain the observed patterns on its own. Other complex multifactorial relationships may select for bacteria harboring AMR, which we hope will be followed up in future studies.

## Data availability statement

The datasets presented in this study can be found in online repositories. The names of the repository/repositories and accession number(s) can be found at: https://www.ncbi.nlm.nih.gov/, PRJNA605147.

## Ethics statement

Ethical review and approval was not required for the animal study because Samples were only collected from the environment of the animals and not direct from the animals. Ethical approval was not sought as sampling from faecal deposits on the ground was deemed outside of the Animal (Scientific Procedures) Act 1986.

## Author contributions

RS, MA, NS, and DR: study design. HM and DG: data collection. MAO, ES, LS, and KC: laboratory sample analysis. RS, HM, MAO, DG, and MV: data analysis. RS, MV, HM, MAO, and MA: manuscript writing. DC and MA: review of study. All authors: review of manuscript. All authors contributed to the article and approved the submitted version.

## Funding

This work was funded by the Antimicrobial Resistance Cross-council Initiative supported by the seven research councils [NE/N019989/1 and NE/N019660/1], and supported by National Institute for Health Research Health Protection Research Unit (NIHR HPRU) in Healthcare Associated Infections and Antimicrobial Resistance at the University of Oxford (NIHR200915) in partnership with Public Health England (PHE). Computation used the Oxford Biomedical Research Computing (BMRC) facility, a joint development between the Wellcome Centre for Human Genetics and the Big Data Institute, supported by Health Data Research UK and the NIHR Oxford Biomedical Research Centre. The publishing of the manuscript was funded by the Veterinary Medicines Directorate under project VM0533. The views expressed are those of the authors and not necessarily those of the NHS, the NIHR, the Department of Health or Public Health England. This work was supported by the NIHR Oxford Biomedical Research Centre. KCC is Medical Research Foundation-funded. DWC, TEAP and ASW are NIHR Senior Investigators. The funders had no role in the design of the study, analyses, interpretation of the data or writing of the manuscript.

## Conflict of interest

The authors declare that the research was conducted in the absence of any commercial or financial relationships that could be construed as a potential conflict of interest.

## Publisher’s note

All claims expressed in this article are solely those of the authors and do not necessarily represent those of their affiliated organizations, or those of the publisher, the editors and the reviewers. Any product that may be evaluated in this article, or claim that may be made by its manufacturer, is not guaranteed or endorsed by the publisher.

## Footnotes

^1^ https://github.com/APHA-AMR-VIR/APHASeqFinder

^2^ https://github.com/tseemann/abricate
