# Supplementary material for: A longitudinal study reveals persistence of antimicrobial resistance on livestock farms is not due to antimicrobial usage alone
Source: Front Microbiol. 2023 Mar 14;14:1070340. doi: 10.3389/fmicb.2023.1070340 (PMC10043416; doi:10.3389/fmicb.2023.1070340)
Supplement: Supplementary file 2 [file Data_Sheet_1.docx]

Appendix Table A: List of known intrinsic antimicrobial resistance (AMR) genes present in *Citrobacter* spp. and *Klebsiella* spp.

| AMR classes | AMR genes |
| --- | --- |
| Aminoglycoside | aac6-Ic |
| Aminoglycoside | aac6-If |
| Aminoglycoside | aph3-Ic |
| Beta lactam | ACT-12 |
| Beta lactam | ACT-15 |
| Beta lactam | ACT-6 |
| Beta lactam | CMY-34 |
| Beta lactam | CMY-37 |
| Beta lactam | CMY-41 |
| Beta lactam | CMY-48 |
| Beta lactam | CMY-53 |
| Beta lactam | CMY-67 |
| Beta lactam | CMY-70 |
| Beta lactam | CMY-77 |
| Beta lactam | CMY-78 |
| Beta lactam | CMY-83 |
| Beta lactam | L-2 |
| Beta lactam | MAL-1 |
| Beta lactam | OXA-12 |
| Beta lactam | SED-1 |
| Beta lactam | SHV-71 |
| Beta lactam | SHV-77 |
| Beta lactam | SRT-1 |
| Beta lactam | ACT-22 |
| Beta lactam | ACT-23 |
| Beta lactam | ACT-27 |
| Beta lactam | ACT-37 |
| Beta lactam | CMY-101 |
| Beta lactam | CMY-51 |
| Beta lactam | CMY-82 |
| Beta lactam | CMY-93 |
| Beta lactam | MIR-8 |
| Beta lactam | OXY-2-10 |
| Beta lactam | OXY-5-2 |
| Beta lactam | SHV-11 |
| Beta lactam | SHV-27 |
| Beta lactam | SHV-40 |
| Beta lactam | SHV-41 |
| Fosfomycin | fosA |
| Quinolone | oqxA10 |
| Quinolone | oqxA11 |
| Quinolone | oqxA3 |
| Quinolone | oqxA6 |
| Quinolone | oqxA8 |
| Quinolone | oqxB10 |
| Quinolone | oqxB12 |
| Quinolone | oqxB13 |
| Quinolone | oqxB20 |
| Quinolone | oqxB24 |
| Quinolone | oqxB25 |
| Quinolone | oqxB26 |
| Quinolone | oqxB32 |
| Quinolone | oqxB4 |
| Quinolone | oqxB5 |
| Quinolone | oqxB9 |
| Quinolone | qnrB10 |
| Quinolone | qnrB23 |
| Quinolone | qnrB27 |
| Quinolone | qnrB4 |
| Quinolone | qnrB50 |
| Quinolone | qnrB51 |
| Quinolone | qnrB56 |
| Quinolone | qnrB6 |
| Quinolone | qnrB7 |
| Quinolone | qnrB8 |
| Quinolone | qnrB40 |

Appendix Table B: Bacterial isolate species detected on each of 14 livestock farms over three sampling visits.

| Farm ID | Animal species | *Citrobacter* spp. | *Escherichia coli* | *Escherichia fergusonii* | *Escherichia* spp. | *Klebsiella* spp. |
| --- | --- | --- | --- | --- | --- | --- |
| RH01 | Pig | 1 | 24 | 7 |  |  |
| RH02 | Pig | 7 | 19 | 7 |  | 3 |
| RH03 | Pig | 1 | 33 | 1 |  |  |
| RH04 | Pig | 2 | 29 | 3 |  | 3 |
| RH06 | Cattle | 6 | 29 | 1 |  |  |
| RH07 | Cattle | 2 | 33 |  |  |  |
| RH08 | Cattle | 1 | 32 | 2 |  |  |
| RH09 | Cattle | 5 | 23 | 3 |  | 4 |
| RH10 | Cattle | 3 | 28 | 2 |  |  |
| RH11 | Sheep | 1 | 33 |  |  |  |
| RH12 | Sheep |  | 29 | 5 |  |  |
| RH13 | Sheep |  | 25 | 8 |  |  |
| RH14 | Sheep |  | 30 | 3 |  | 1 |
| RH15 | Sheep | 2 | 22 | 9 | 1 |  |

Appendix Table C: List of antimicrobial resistance (AMR) genes and classes identified in bacterial isolates collected from 14 livestock farms.

| AMR Class | AMR genes | Number of isolates harbouring AMR gene |
| --- | --- | --- |
| Amino | *aac3-IId* | 1 |
| Amino | *aac3-IVa* | 26 |
| Amino | *aadA2* | 35 |
| Amino | *aadA5* | 2 |
| Amino | *ant3-1a* | 26 |
| Amino | *ant3-Ia (long)* | 37 |
| Amino | *aph3-Ia* | 20 |
| Amino | *aph3-IIa* | 18 |
| Amino | *aph4-Ia* | 26 |
| Amino | *aph3-Ib (strA)* | 63 |
| Amino | *aph6-Id (strB)* | 63 |
| non-ESC | *bla*_CMY-104_ | 6 |
| non-ESC | *bla*_LAP-2_ | 1 |
| non-ESC | *bla*_TEM-1b_ | 54 |
| non-ESC | *bla*_TEM-1c_ | 5 |
| non-ESC | *bla*_TEM-1d_ | 1 |
| ESC | *bla*_CMY-66_ | 1 |
| ESC | *bla*_CTX-M-14_ | 1 |
| ESC | *bla*_SHV-145_ | 1 |
| MLS | *lnuF* | 1 |
| MLS | *mefB* | 1 |
| MLS | *mphA* | 6 |
| MLS | *mphB* | 7 |
| Phen | *catA1* | 1 |
| Phen | *cmlA1* | 35 |
| Quino | *parC* (QRDR) | 1 |
| Quino | *qnrB10* | 1 |
| Quino | *qnrB19* | 1 |
| Quino | *qnrB35* | 1 |
| Quino | *qnrD* | 1 |
| Quino | *qnrS1* | 2 |
| Strep | *sat2A* | 7 |
| Sulph | *sul2* | 45 |
| Sulph | *sul3* | 32 |
| Sulph | sul3 (v1) | 39 |
| Tetra | *tet(A)B* | 59 |
| Tetra | *tet(A)** | 69 |
| Tetra | *tet(M)* | 3 |
| Trim | *dfrA1* | 10 |
| Trim | *dfrA12* | 20 |
| Trim | *dfrA14* | 20 |
| Trim | *dfrA16* | 1 |
| Trim | *dfrA17* | 2 |
| Trim | *dfrA36* | 2 |
| Trim | *dfrA5* | 2 |
| Trim | *dfrA8* | 1 |

AMR class abbreviations: Amino = aminoglycoside, ESC = ESC beta-lactam, non-ESC = non-ESC beta-lactam, MLS = Macrolides, Lincosamides, and Streptogramins, Phen = Phenicol, Quino = Quinolone, Strep = Streptothricin, Sulph = Sulphonamide, Tetra = Tetracycline, Trime = Trimethoprim. *Tet(A) includes variants v1 (1 isolate), v4 (65), v6 (1) and v9 (2).

Appendix Table E: Summary of antimicrobial class usage (in mg of active ingredient/ kg of treated animals) per farm per sampling visit. This table does not include study farms RH13 and RH15 which did not use any antimicrobial during the study.

| Farm ID | Visit No | Aminocoumarin | Aminoglycosides | ESC Beta Lactams | MLS | Non-ESC Beta Lactams | Pleuromutilin | Quinolones | Sulphonamide | Tetracyclines | Trimethoprim |
| --- | --- | --- | --- | --- | --- | --- | --- | --- | --- | --- | --- |
| RH01 | 1 |  | 18.6 |  | 93.1 | 32.5 |  | 7.5 | 699.9 | 48.2 | 140.0 |
| RH01 | 2 |  | 10.0 | 5.0 | 0.3 | 189.3 | 48.5 | 6.0 | 12.3 | 15.0 | 2.5 |
| RH01 | 3 |  | 10.0 |  | 6.5 | 420.6 |  | 13.6 |  |  |  |
| RH02 | 1 |  | 28.6 |  | 139.5 | 44.1 |  | 7.5 |  | 143.1 |  |
| RH02 | 2 |  | 893.4 |  | 24.2 | 44.8 |  | 7.6 | 88.8 |  | 17.8 |
| RH02 | 3 |  | 1,818.2 |  | 74.9 | 210.5 |  | 7.5 | 77.5 |  | 15.5 |
| RH03 | 1 |  |  |  |  |  |  |  |  |  |  |
| RH03 | 2 |  |  |  |  |  |  |  |  |  |  |
| RH03 | 3 |  |  |  | 83.1 |  |  |  |  |  |  |
| RH04 | 1 |  |  |  | 8.0 | 24.0 |  |  |  |  |  |
| RH04 | 2 |  |  |  | 6.2 | 60.1 |  |  |  |  |  |
| RH04 | 3 |  |  |  | 38.9 | 15.0 |  |  |  |  |  |
| RH06 | 1 |  | 60.3 |  |  | 223.7 |  |  |  |  |  |
| RH06 | 2 |  | 36.9 |  |  | 213.1 |  |  |  |  |  |
| RH06 | 3 |  | 33.4 |  |  | 76.8 |  |  |  |  |  |
| RH07 | 1 | 0.5 | 31.6 | 3.2 | 11.8 | 32.4 |  |  |  |  |  |
| RH07 | 2 |  | 66.1 | 5.1 |  | 55.9 |  |  |  |  |  |
| RH07 | 3 |  | 45.5 | 10.3 | 47.1 | 52.7 |  | 21.8 |  |  |  |
| RH08 | 1 |  |  | 10.6 |  |  |  | 0.6 |  | 70.6 |  |
| RH08 | 2 |  | 274.5 | 14.4 | 2.6 | 215.4 |  |  |  |  |  |
| RH08 | 3 |  | 60.3 | 4.9 | 37.6 | 51.7 |  |  |  |  |  |
| RH09 | 1 |  | 113.7 | 18.7 | 122.4 | 166.3 |  | 14.1 | 150.6 | 53.6 | 30.1 |
| RH09 | 2 | 0.9 | 70.6 | 3.6 | 21.2 | 85.2 |  |  | 54.1 |  | 10.8 |
| RH09 | 3 | 0.2 | 66.4 | 3.0 |  | 64.4 |  |  | 58.8 | 30.1 | 16.1 |
| RH10 | 1 | 2.1 | 19.3 | 8.6 | 39.0 | 21.5 |  | 8.0 |  | 10.6 |  |
| RH10 | 2 | 0.7 | 13.1 | 7.2 | 6.7 | 9.8 |  |  |  | 10.0 |  |
| RH10 | 3 | 0.7 | 13.1 | 7.2 | 35.9 | 9.8 |  |  |  | 4.4 |  |
| RH11 | 1 |  |  |  |  |  |  |  |  |  |  |
| RH11 | 2 |  |  |  |  |  |  |  |  |  |  |
| RH11 | 3 |  | 10.0 |  |  | 8.0 |  |  |  |  |  |
| RH12 | 1 |  |  |  |  |  |  |  |  | 1.9 |  |
| RH12 | 2 |  | 13.6 |  |  | 8.9 |  |  |  | 56.3 |  |
| RH12 | 3 |  |  |  |  |  |  |  |  | 63.6 |  |
| RH14 | 1 |  |  |  |  |  |  |  |  | 13.3 |  |
| RH14 | 2 |  |  |  |  | 15.0 |  |  |  | 40.0 |  |
| RH14 | 3 |  |  |  |  |  |  |  |  | 76.7 |  |

ESC = extended spectrum cephalosporins. MLS = macrolides, lincosamides, and streptogramins


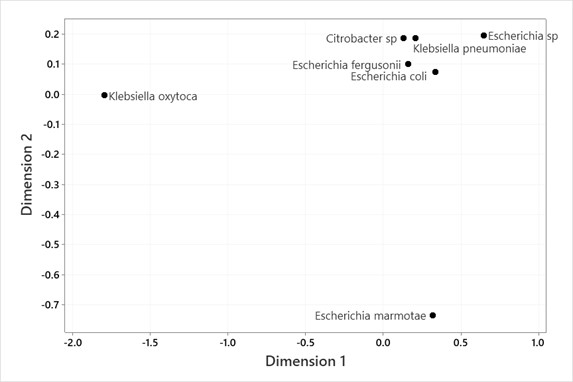


Appendix Figure A: Multi-dimensional scaling plot indicating the similarity of AMR genotype profiles amongst bacterial species cultured from samples from 14 livestock farms (results summarised from percentage of isolates resistant to each antimicrobial (n=484)). The summarised AMR results from each bacterial species are scaled to two dimensions (x and y axis) to present the similarity between species. Results from Escherichia marmotae, ‘other Escherichia spp’ and Klebsiella oxytoca should be treated with caution as they represent only a single isolate each.


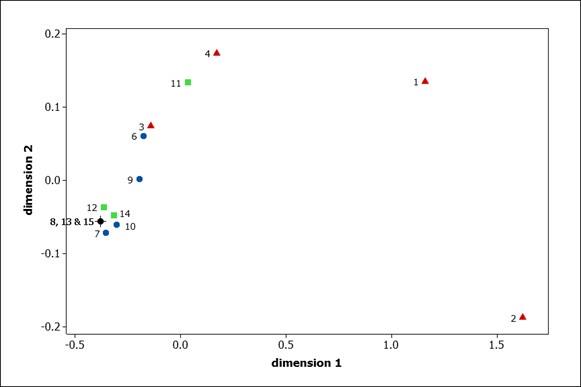


Appendix Figure B: Multi-dimensional scaling plot indicating the similarity of AMR genotype profiles in isolates cultured from 14 livestock farms (labelled with their unique farm “RH” identifier number). The results were summarised from the percentage of faecal Enterobacterales isolates resistant to each antimicrobial (n=484). The summarised AMR results from each bacterial species are scaled to two dimensions (x and y axis) to present the similarity between species. Red triangles indicate pig farms, blue dots are cattle farms and green squares are sheep farms. Cattle farm 8 and sheep farms 13 and 15 occupied the same position.

Appendix Figure C: Comparison of proportion of aminoglycoside resistant isolates (Amino % AMR; represented by bars) and aminoglycoside usage (mg/kg of treated animals; represented by dots) on five farms (RH01-RH09) which demonstrated increasing or decreasing trends of resistance to at least one antimicrobial class across their three sampling visits.

Appendix Figure D: Comparison of proportion of beta-lactam resistant isolates (BetaL % AMR; represented by bars) and beta-lactam usage (ESC BetaL and Non-ESC BetaL in mg/kg of treated animals; represented by dots) on five farms (RH01-RH09) which demonstrated increasing or decreasing AMR trends to at least one antimicrobial class across three sampling visits.

Appendix Figure E: Comparison of proportion of MLS (macrolides, lincosamides, and streptogramins) resistant isolates (MLS % AMR; represented by bars) and MLS usage (mg/kg of treated animals; represented by dots) on five farms (RH01-RH09) which demonstrated increasing or decreasing AMR to at least one antimicrobial class across three sampling visits.

Appendix Figure F: Comparison of proportion of trimethoprim resistant isolates (Trim % AMR; represented by bars) and trimethoprim usage (mg/kg of treated animals) on five farms (RH01-RH09) which demonstrated increasing or decreasing AMR trends to at least one antimicrobial class across three sampling visits.

Appendix Figure G: Comparison of proportion of tetracycline resistant isolates (Tetra % AMR; represented by bars) and tetracycline usage (mg/kg of treated animals) on five farms (RH01-RH09) which demonstrated increasing or decreasing AMR trends across three sampling visits.

Appendix Figure H: Proportion of aminoglycoside and sulphonamide resistant isolates (Amino and Sulph % AMR) and antimicrobial usage (in mg/kg of treated animals) on a cattle farm (ID RH07) over three sampling visits.

Appendix Figure I: Proportion of tetracycline resistant isolates (Tetra % AMR) and antimicrobial usage (in mg/kg of treated animals) on a sheep farm (ID RH12) over three sampling visits.

Appendix Figure J: Proportion multi-drug resistant (MDR) isolates and antimicrobial usage (in mg/kg of treated animals) on a cattle farm (ID RH10) over three sampling visits.
